# Emulgel Containing Metronidazole and Clindamycin for the Treatment of Rosacea

**DOI:** 10.3390/pharmaceutics17020168

**Published:** 2025-01-27

**Authors:** Guillermo De Grau-Bassal, Ana Cristina Calpena-Campmany, Marcelle Silva-Abreu, Joaquim Suñer-Carbó, Mireia Mallandrich-Miret, Sergio Martínez-Ruiz, Cecilia Cordero, Alfonso Del Pozo, Núria Bozal-de Febrer

**Affiliations:** 1 Departament de Biologia, Sanitat i Medi Ambient, Facultat de Farmàcia i Ciències de l’Alimentació, Universitat de Barcelona (UB), 08028 Barcelona, Spain; gdegrau@ub.edu (G.D.G.-B.); nuriabozaldefebrer@ub.edu (N.B.-d.F.); 2 Department of Pharmacy, Pharmaceutical Technology and Physical Chemistry, Faculty of Pharmacy and Food Sciences, University of Barcelona, 08028 Barcelona, Spain; anacalpena@ub.edu (A.C.C.-C.); jsuner@ub.edu (J.S.-C.); mireia.mallandrich@ub.edu (M.M.-M.); apozo@ub.edu (A.D.P.); 3 Institute of Nanoscience and Nanotechnology (IN2UB), University of Barcelona, 08028 Barcelona, Spain; 4 Departament de Bioquímica i Fisiologia, Facultat de Farmàcia i Ciències de l’Alimentació, Universitat de Barcelona, 08028 Barcelona, Spain; sergio_martinez_ruiz@ub.edu (S.M.-R.); corderocecilia16@gmail.com (C.C.)

**Keywords:** rosacea, clindamycin, metronidazole, emulgel

## Abstract

Rosacea is a common skin condition with quite a relevance. It currently affects at least 10% of the European population at some point after the age of 30. It is a chronic disorder that mainly affects the skin on the face and is characterized by outbreaks and remissions. Under normal circumstances, the skin face presents a wide range of commensal organisms, such as *Staphylococcus epidermidis* or *Demodex folliculorum*, but dysbiosis of the skin flora plays a relevant role in inflammatory processes and the development of the disease. Metronidazole (MD) is one of the main treatments indicated to reduce redness on the cheeks, nose, chin, or forehead and also to treat flushing, erythema, pimples, and other symptoms due in part to its anti-inflammatory action. On the other hand, clindamycin (CM) is another antibiotic used for rosacea, especially for its action against anaerobic and Gram-positive bacteria. **Background/Objectives**: This study aimed to develop an emulgel formulation that includes MD and CM, using excipients with non-comedogenic and non-irritating properties. **Methods**: The formulation was characterised physiochemically, rheological measurements were made, and short-term stability studies were carried out. In vitro release, permeation studies, toxicity an in vitro inflammation model were evaluated in a HaCaT cell model. To determine the interaction between the antibiotics, the minimum inhibitory concentration was determined separately and together using the broth microdilution method. To determine the formulation’s antimicrobial activity, an agar diffusion method was used. **Results:** The MD-CM-gel droplet size was measured by laser diffraction and the diameter obtained was less than 2.68 ± 0.18 µm in 50% of the particles. Suitable results was observed for the short-term stability. Release and permeation data revealed sustained drug release and adequate permeation through human skin. Non-toxicity was detected and the MD showed an anti-inflammatory effect with non-interference of CM. Also, there is no antagonism between the two antibiotics and the MD-CM-gel shows better results when compared to the formulations with the antibiotics separately and to commercial formulations. **Conclusions**: It is suggested that, following detailed preclinical and clinical studies, MD-CM-gel could be considered as an alternative for treating rosacea.

## 1. Introduction

Rosacea is a chronic inflammatory skin disease that affects an estimated 5% to 46% of the adult population, particularly those over 30 years of age [1]. Redness, papules, pustules, telangiectasias, flushing episodes, and erythema are some of the symptoms that can appear, particularly in the central area of the face. It is common for an individual to present multiple symptoms simultaneously [2,3,4]. Rosacea is classified into different subtypes based on these symptoms: erythematotelangiectatic rosacea, papulopustular rosacea, phymatous rosacea, and ocular rosacea [5,6]. The exact cause of rosacea remains unclear; however, several factors are considered potential contributors. These include alcohol consumption, certain medications or foods, sun exposure, emotional stress, chronic vasodilation, and inflammatory changes [7]. Additionally, the presence of microorganisms, such as certain skin commensal bacteria and the mite Demodex follicularum, has been noted in rosacea patients [8]. This disease affects the skin and can lead to dysbiosis, which can increase the population of certain microorganisms [9]. It is not clear that microorganisms are the cause of rosacea. However, some of these, such as *Stahpyloccocus epidermidis* or *Cuticubabacterium acnes*, can activate the immune system via Toll-like receptors [10].

Treatments usually include anti-inflammatories, skin care, sunscreens, retinoids, antibiotics, laser treatments, and surgical techniques. The type of rosacea that is impacted determines which therapies are appropriate. Erythematotelangiectatic and papulopustular rosacea are usually treated with topical treatments, such as metronidazole (MD) or azelaic acid. In contrast, others such as phymatosa are treated orally or with lasers or electrosurgery; in the case of ocular rosacea, the treatment is mainly with artificial tears [11]. Moreover, topical treatments to reduce the inflammation or the intensity of symptoms, such as MD and other topical antibiotics, are also being used, including clindamycin (CM) [12].

MD is a synthetic antibiotic derived from azomycin and produced by bacteria of the genus Actinobacteria and Proteobacteria. It can be administered in various ways, intravenously, vaginally, or orally, although the topical route is the most used to treat rosacea [13]. Topical MD is effective in reducing the symptoms of rosacea, especially when administered at 0.75% in formulations with a high water content, such as creams and gels [14]. Topical treatment with MD was first used in the 1980s and it was observed that its effect was due to its ability to reduce reactive oxygen species produced by neutrophils during inflammation [15,16].

CM is another antibiotic used in the treatment of rosacea. It is powerful in lowering symptoms, and its effect, like different antibiotics, is unclear. It may be due to a direct effect on microorganisms, or it may be related to the reduction in inflammation. In these treatments, increased irritation has been observed in some cases, probably due to the components for its delivery [17,18].

Ointments, creams, lotions, and gels are some examples of topical dosage forms. These have many disadvantages, such as stickiness, stability problems, and poor spreadability; they could also cause allergic reactions and sometimes show poor permeability and absorption [19]. However, emulsions are easy to remove, spreadable, thixotropic, non-greasy, have a pleasant appearance, are emollient, and have a long shelf life. Nowadays, emulgels are used to deliver many drugs, such as analgesics, anti-inflammatories, anti-acne, and antifungals. It is therefore of great pharmacological importance and is relatively free of side effects [20].

Emulgels are an emerging topical drug delivery system that combines the properties of emulsions and gels. For rosacea, it can be advantageous since it allows the incorporation of both hydrophilic and hydrophobic drugs, potentially improving the effectiveness of treatments. They also tend to have better stability and patient compliance compared to traditional creams or ointments [21].

This is the first type of study that investigates the efficacy of MD and CM in the treatment of rosacea. Therefore, this study aims to develop an emulgel formulation incorporating MD and CM, along with mild excipients, emollients, and non-comedogenic agents. The formulation undergoes physicochemical characterization, evaluation of biopharmaceutical properties, and assessment of toxicity and efficacy in vitro using a HaCaT cell line inflammation model, as a potential treatment for rosacea.

## 2. Materials and Methods

### 2.1. Materials

MD and CM were purchased from Acofarma (Barcelona, Spain); glycerine was obtained from Comercial Química Massó (Barcelona, Spain); Simulgel^®^ 600 (acrylamide/sodium acryloyldimethyl taurate copolymer and isohexadecane and polysorbate 80) was acquired from SEPPIC (Madrid, Spain); MCT (medium-chain triglycerides) were purchased from Fagron (Terrassa, Spain); isohexadecane was supplied by Inquiaroma (Barcelona, Spain); Phenotip^®^ (phenoxyethanol (and) methylparaben (and) ethylparaben (and) propylparaben (and) butylparaben (and) isobutylparaben) was obtained from Clariant (Leeds, UK). Potassium dihydrogen phosphate, methanol, and acetonitrile were supplied by Panreac (Madrid, Spain). Muller-Hinton broth, Muller-Hinton agar, tryptone soya broth, tryptona soya agar, and yeast extract powder were obtained from Oxoid Sigma-Aldrich (Madrid, Spain). Reagents used in the analytical method and sample analysis were acquired from Panreac (Barcelona, Spain); a Millipore Milli-Q purification system (Millipore Corporation, Burlington, MA, USA) provided the purified water used in this work. All other chemicals and reagents used in the study were of analytical grade. Nylon membranes, with a molecular weight cutoff at 12,000–14,000 Da, were acquired from Iberlabo (Madrid, Spain).

### 2.2. Emulgel Preparation

Following the recommendations of the clinical practice in the association of MD to topical antibiotics, an emulgel was developed containing the combination of MD and CM. The composition of the emulgel is shown in Table 1.

**Figure 1:**
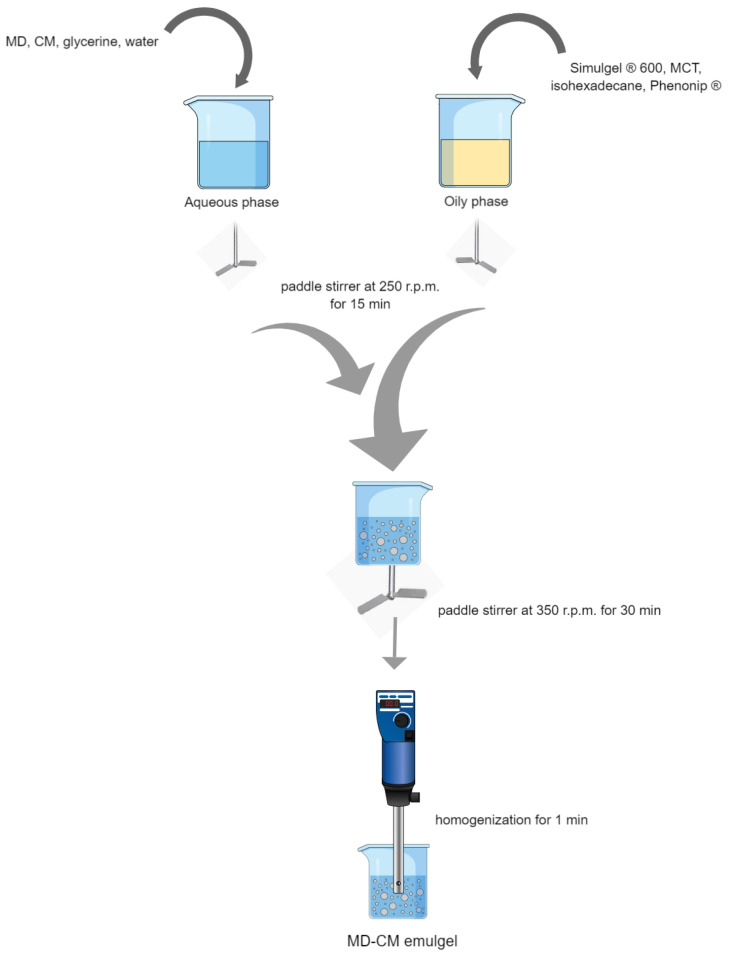
Schematic representation of the preparation of the MD-CM-gel, consisting of an aqueous phase and an oily phase that were combined, mixed, and homogenized.

The emulgel consists of two phases: The aqueous phase was prepared by adding MD and CM in water and glycerine, and then, the solution was mixed with a paddle stirrer at 250 r.p.m. for 15 min. Separately, the oily phase was composed of Simulgel^®^ 600, MCT, isohexadecane, and Phenonip^®^. The ingredients were mixed in a paddle stirrer at 250 r.p.m. for 15 min. The aqueous phase was added slowly to the oily phase, avoiding air formation in the emulsion. Then, it was mixed with the paddle stirrer for 30 min at 350 r.p.m. And finally, it was homogenized by Ultra-Turrax T50- (Ika Laborteknik, Staufen, Germany) at the lowest speed for 1 min to form the emulgel (MD-CM-gel). A free-drug emulgel was also prepared for microbiological study, as mentioned above. Figure 1 depicts the preparation of MD-CM-gel.

### 2.3. Physicochemical Characterization of the Emulgel

#### 2.3.1. Organoleptic Characteristics

The product should be white, bright with a uniform texture, and lump-free. The emulgel was visually observed for phase separation, creaming, exudate, colour changes, or any sign of destabilization.

#### 2.3.2. Stability Study and Optical Microscopy

Several aliquots of the product were stored in 100 mL Unguator^®^ flasks for the stability study. During time 0, 3, and 6 months, the storage conditions were room temperature 25 ± 2 °C. These samples were used to evaluate the visual appearance, and pH was measured by the pH meter CRISON micro-pH 2000 (Crison Instruments S.A., Alella, Spain).

The morphology of emulgel formulation was observed by a microscope (Model DM 1000 LED; Leica Microsystems) with image acquisition (Model EC; Leica Microsystems). The droplet size should be below 20 µm diameter.

#### 2.3.3. Surface Spreadability

The surface spreadability of the emulgel was assessed as the increase in the surface of a given amount of the formulation when increasing weights were applied at defined time points. The spreadability was conducted by an extensometer (26). On 0.35 g of formulation, the following weights were applied for 1 min: 26.06; 36.06; 46.06; 76.06; 126.06; 146.06; 176.06, and 226.06 g. Measurements were conducted after freshly prepared emulgel. The expansion in diameter was recorded as a function of the weight applied. Each measurement was estimated according to Equation (1):

(1) $$S=d^{2}\times \frac{\pi }{4}$$

where *S* is the surface spreading area (mm^2^) computed from the utilised mass of sample (g), and d is the average diameter (mm) impacted by the sample. Experimental results were analysed according to the best kinetic model and fitted to different mathematical models with the statistical program using GraphPad Prism^®^ 10.2.3. The *r* value confirmed the model fitting.

#### 2.3.4. Droplet Size Measurement 

The droplet size was determined by Mastersizer^®^ 2000 (Malvern Instruments, Worcestershire, UK) using laser diffraction at 25 °C. Milli-Q water was used as a dispersant, yielding the relative distribution of the volume of droplets. The range of size classes was determined according to the Mie theory. Measurements were collected at room temperature (mean ± SD, n = 3) over time up to 6 months. The results were processed by the software Mastersizer^®^ 2000 version 5.6 (32).

#### 2.3.5. Rheological Behavior

Rheological rotational measurements were performed using a Haake Rheostress^®^ 1 rheometer (Thermo Fisher Scientific, Karlsruhe, Germany). For all of measurements, the device was connected to the thermostatic circulator Thermo^®^ Haake Phoenix II + Haake C25P and a computer PC provided with Haake Rheowin^®^ Job Manager v. 4.91 software (Thermo Electron Corporation, Karlsruhe, Germany) to execute the tests and Haake Rheowin^®^ Data Manager v. 4.91 software (Thermo Electron Corporation, Karlsruhe, Germany) to perform the analyses of the obtained data. Steady-state measurements were addressed with cone-and-plate geometry (0.105 mm gap) with a fixed lower plate and a mobile upper cone Haake C60/2° Ti (60 mm diameter, 2° angle). The shear stress (τ) was measured as a function of the shear rate (γ). Viscosity curves (η = f(γ)) and flow curves (τ = f(γ ·)) were recorded at 25 ± 0.1 °C. The shear rate ramp program included a 3 min upward flow curve period from 0.1 to 100 s^−1^, a 1 min constant shear rate period at 100 s^−1^, and finally, a 3 min downward flow curve period from 100 to 0.1 s^−1^. Data from the flow curves were fitted using mathematical models to identify the model that provided the best overall match of the experimentally observed rheological data: Bingham, Ostwald de Waele, Herschel-Bulkley, Casson, and Cross. The adequacy of the rheological profiles to the mathematical models was based on the correlation coefficient value (r) and chi-square value. The determination of the disturbance of the microstructure during the test or apparent thixotropy (Pa/s) was evaluated by the determination of the area of the hysteresis loop. Steady-state viscosity (η, Pa.s) was determined from the constant share section at 100 s^−1^. Analyses of samples were carried out at 25 ± 0.2 °C, 24 h, 3 months, and 6 months after preparation.

### 2.4. Infrared Interaction Study

To obtain FTIR spectra of MD, CM, blank gel, and the emulgel formulation, a Thermo Scientific Nicolet iZ10, with an ATR diamond and DTGS detector, was used. The scanning range was 525–4000 cm^−1^.

### 2.5. In Vitro Drug Release

The drug release was investigated by vertical diffusion Franz cells (Microette Hanson Research^®^) at 32 °C with a diffusional area of 0.64 cm^2^, and a nylon membrane with 0.45 µm pore size was used. The receptor fluid was Milli-Q water/DMSO (80:20), which was continuously stirred at 324 rpm. A total of 250 mg of emulgel was applied to the membrane (n = 6), and aliquots of 300 µL were withdrawn at the sampling times: 0.16 h, 1 h, 3 h, 5.3 h, 8.1 h, 9.5 h, and 23.3 h. Following sample collection during the in vitro release tests, all samples were promptly stored at 4 °C in a refrigerator to maintain their stability and integrity. Sample analysis was performed immediately after completing the release test to ensure accurate and reliable quantification of the active ingredient. Samples were quantified separately by HPLC (High-Performance Liquid Chromatography).

The cumulative released amounts of each drug were plotted vs. time, and the kinetic profile of the drugs was determined by fitting the data to mathematical models and the goodness-of-fit was based on the best determination coefficient (*r*^2^).

### 2.6. Ex Vivo Permeation

The permeability of the drugs was evaluated on human skin by vertical Franz diffusion cells. This study was carried out by the Hospital de Barcelona Ethics Committee (dated 17 January 2020); this study utilized human skin obtained from a healthy 38-year-old woman who underwent an abdominal lipectomy at Hospital de Barcelona, SCIAS, Barcelona, Spain, with prior written consent. The study was conducted as described in Section 2.5 but using ex vivo human skin (n = 6) instead of the synthetic membrane. A total of 250 mg of emulgel was applied to the skin, and 300 µL were collected from the receptor medium at the sampling time points of 1 h, 5.5 h, 9.75 h, 23.4 h, 27.2 h, and 31.1 h and replaced with fresh receptor medium MiliQ water and DMSO (80:20). Receptor fluid samples were stored at 4 °C in a refrigerator immediately after collection to maintain stability until analysis. At the end of the assay, the drugs retained in the skin were extracted by adding 1 mL of solvent (water) to the skin and sonicated for 20 min. The supernatants were collected for further analysis. The samples of drug extraction from the skin were analysed immediately after collection, alongside the receptor fluid samples that had been kept in the refrigerator. Quantitative determination of the permeated MD and CM were analysed in triplicate by HPLC. Kinetic parameters were estimated using GraphPad Prism^®^ 10.2.3) (GraphPad Software Inc., San Diego, CA, USA).

For the collected samples, the cumulative amount of MD and CM (μg) permeated per cm^2^ of skin was plotted against time (h). The permeation profile was analysed based on the diffusion model for infinite dose conditions. The MD and CM flux through the skin ((*Jss*, g/(h)) was calculated by plotting the cumulative amount of drug permeated through the skin versus time, and the slope of the linear part of the curve was determined by linear regression analysis using GraphPad Prism^®^ 10.2.3 divided by the diffusion area. The lag time (Tl, h) was determined as the X-intercept of the linear regression. The permeation coefficient (*Kp*, cm/h) was determined by dividing *Jss* by the initial drug concentration (*Co*, µg/mL) in the donor compartment. The partition and diffusion coefficients were estimated according to Equations (2) and (3):

P2 = 1/6 × Tl (2)

where Tl is the lag time previously determined as the X-intercept.

P1 = Kp/P2 (3)

where Kp is the permeability coefficient, and P2 is the partition coefficient.

The steady-state plasma concentration (Css) of the drug passing through the skin barrier after topical application was determined using the following Equation (4):

*Css* = *Jss* × *A*/*Clp* (4)

where *Css* is the steady-state plasma concentration, *Jss* is the flux, *A* is the area of application, and *Clp* the plasma clearance.

### 2.7. Analytical Method

The samples from the release and the permeation assays were analysed by HPLC (Alliance 2695 (A1.2) Waters) with a PDA detector (Waters 2996 (138C)). The column used was an ODS Hypersil^®^ 250 × 4 mm, 5 µm Thermo. The analytical conditions for the determination of MD and CM are given in Table 2.

### 2.8. Antibacterial Activity

#### 2.8.1. Bacterial Strains and Culture Conditions

For this study, microorganisms present in the oily area of the skin, follicle, and sebaceous glands were selected. An *Escherichia coli* strain was also included as a model of Gram-negative bacillus. Strains were obtained from different culture collections: *Staphylococcus epidermidis* ATCC 12228, *Staphylococcus aureus* ATCC 29213, *Micrococcus luteus* ATCC 10240, *Cutibacterium acnes* ATCC 6919, *Corynebacterium tuberculostearicum* CECT 763 (ATCC 33035), *Corynebacterium simulans* DSM 44415, *Staphylococcus capitis* CECT 7101^T^ (T = Type strain), *Staphylococcus hominis* CECT 234^T^, *Streptococcus mitis* DSM 12643, and *Escherichia coli* ATCC 25922.

*E. coli*, *S. aureus, S. epidermidis*, *and M. luteus* were incubated on Muller-Hinton (MH) agar at 37 °C in aerobic conditions. *C. simulans, S. mitis, S. capitis*, and *S. hominis* were incubated on agar plates prepared with Tryptic soy broth (TSB) + 0.3% yeast extract + 1.5% agar at 37 °C in aerobiosis. *C. tuberculostearicum* was incubated on agar plates with TSB + 0.1% tween 80 + 1.5% agar at 37 °C in aerobic conditions and *C.acnes* was incubated on MH agar plates at 37 °C in anaerobiosis. The same media have been used without agar for the microdilution method.

#### 2.8.2. Antimicrobials Interactions

Interactions between CM and MD have been determined by calculating the minimum inhibitory concentration (MIC) of each antibiotic separately and combinations of both. Assays were performed using the standardised broth microdilution method [22]. Stock solutions of antibiotics were prepared in the broth culture indicated for each microorganism. For stock solutions, MD and DMSO were added, never exceeding 5% of the solvent. To study the interactions between the two antimicrobials, the microdilution plate was filled with 100 µL of doubly concentrated antibiotic. MD was distributed on the ordinate axis and CM was on the abscissa axis. Controls of the uninoculated culture medium, strain growth, and uninoculated antibiotic solutions were incorporated into the wells of the microdilution plates.

The strains used in the assay were incubated under optimal conditions, as mentioned in the previous section. Suspensions of 1 × 10^8^ CFU/mL were prepared using a spectrophotometer (UV-1800 Simadzu UV Spectrophotometer) at 600 nm. The wells of the microdilution plate were inoculated with 10 µL of a 1/10 dilution of the microorganism suspension. At 48 h of incubation, a visual reading of the growth and a reading of the optical density was made by spectrophotometer (microplate Reader Synergy|HT, Biotek, Winooski, VT, USA).

Growth was calculated by determining the percentage growth of each of the antibiotic combinations concerning the positive control using the following equation:

*(ODp − ODp C−)/(OD C + OD C−)* × 100 (5)

*ODp* is the optical density of the well of one of the combinations of antibiotics; *ODp C−* is the optical density of the same combination without inoculation; *OD C+* is the optical density of the positive growth control of that microorganism; and *OD C−* is the optical density of the culture medium used without inoculation. Growth was considered to have occurred after a 10% increase for the positive control.

#### 2.8.3. Antimicrobial Efficacy

The antimicrobial activity of formulations was tested by using a modified disk diffusion test agar [23], measuring the inhibition areas produced. The same microorganisms and culture conditions were used as described above, except for *S. mitis* due to the difficulty of surface growth, which has made it difficult to read the results.

Suspensions of the different microorganisms were prepared at a concentration of 0.5 on the McFarland scale. The strains were planted confluently in the appropriate medium with the aid of a soaked swab and rotating the plate at approximately 60 °C between each inoculation (three times) to ensure an even distribution of the inoculum. A total of 50 µL from the formulation with the two antimicrobials, as well as from the formulations with only MD, only CM, and without antimicrobials, was added. The same procedure was used for two solid commercial formulations: commercial formulation 1 (CO1) with 1% clindamycin and commercial formulation 2 (CO2) with 0.75% metronidazole. No more than three formulations per plate were placed and incubated under the optimal conditions for each microorganism described previously. Once the incubation time had elapsed, the inhibition halos were measured for each formulation and microorganism.

### 2.9. Toxicity

Human keratinocytes (HaCaT) cells were cultured as described previously [24]. Cells were grown in high glucose DMEM (Dulbecco’s Modified Eagle’s Medium (Thermofisher)), supplemented with 10% fetal bovine serum (FBS), 2 mM l-glutamine, 100 units/mL penicillin G, and 100 µg/mL streptomycin. Cells were incubated at 37 °C in an atmosphere of 5% CO2. For cytotoxicity assays, cells were grown at 80–90% of confluence.

Cytotoxicities of MD and CM were determined by the MTT (3-(4,5-Dimethylthiazol-2-yl)-2,5-diphenyl tetrazolium bromide) assay, which measures the reduction of tetrazolium salt by intracellular dehydrogenases of viable living cells. Compounds were tested at concentrations indicated in the results section. For this, HaCaT cells were seeded in 96-well plates with 100 μL of culture medium (DMEM) at a density of 2 × 10^4^ cells/well. When cells reached 90% confluency, cells were incubated with each compound or a mixture of both for 24 h. Then, the medium was removed and MTT (Sigma-Aldrich Chemical Co., St. Louis, MO, USA) was added at 0.25% in PBS. After a 3 h incubation, the medium was replaced by 100 µL DMSO (99% dimethyl sulfoxide, Sigma-Aldrich). Cell viability was then determined at a wavelength of 570 nm in a Modulus^®^ Microplate Photometer (Turner BioSystems Inc., Sunnyvale, CA, USA). Results were expressed as a percentage of cell survival relative to untreated cells. Since CM is not soluble in water, DMSO has been used as a solvent. For this reason, the cytotoxicity studies have included the analysis of the effect of DMSO present in the CM solutions after the corresponding dilutions.

The results were expressed as the percentage of cell survival relative to the control (untreated cells). See Equation (6).

Cell viability = ABS treated cells/ABS control cells × 100 (6)

### 2.10. Inflammation Activity

The in vitro model of inflammation used in this study involved HACAT cells exposed to LPS-IFNγ at 0.5 µg/mL and 0.02 µg/mL. Cells were incubated with MD and/or CM for 3 h before the addition of LPS-IFNγ. In parallel, cells stimulated only with LPS-INFγ were set as a positive control and untreated cells as a negative control [25]. Following 48 h incubation, IL-6 secreted levels were quantified in the culture supernatant using the enzyme-linked immunosorbent assay (ELISA) (BD Biosciences, San Jose, CA, USA), according to the manufacturer’s instructions. Data were representative of 3 independent experiments.

### 2.11. Data Analysis

The statistical analysis of the permeation studies was conducted using GraphPad Prism^®^ 10.2.3 (GraphPad Software Inc., San Diego, CA, USA). The values were expressed as averages ± SEM. The software packages Haake RheoWin^®^Job Manager V.3.3 and RheoWin^®^Data Manager V.3.3 (Thermo Electron Corporation, Karlsruhe, Germany) were used to carry out the testing and analysis of the obtained rheological data, respectively.

## 3. Results

### 3.1. Characterization of the Emulgel

**Figure 2:**
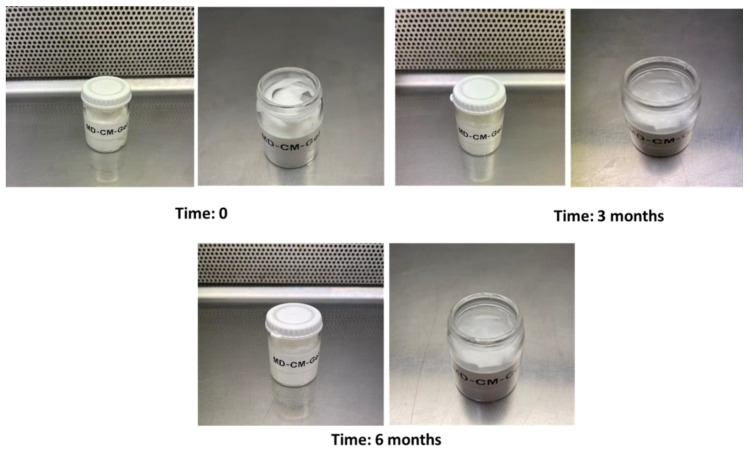
Visual observation of the MD-CM-gel at time 0, 3, and 6 months after its preparation.

**Figure 3:**
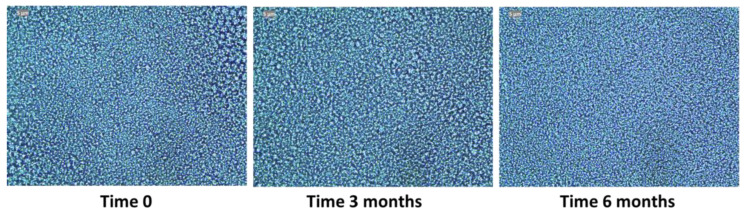
Microscopy images of MD-CM-gel at time 0, 3, and 6 months after its preparation.

Once the emulgel was prepared, it showed a white colour, bright with uniform texture, lump-free, and no separate phase was detected. This was determined at time 0, 3, and 6 months after its preparation and did not show visual changes over time (Figure 2). Moreover, the morphology determination by microscopy of the MD-CM-gel showed dispersed and uniform particles over 6 months (Figure 3). The facial pH in rosacea patients is slightly acidic compared to the normal facial skin. So, the emulgel should have a pH between 5.5 and 6.5 (28). The pH was monitored at times 0, 3, and 6 months and no changes were observed during this period, the pH was stable, and the values are shown in Table 3.

### 3.2. Extensibility

**Figure 4:**
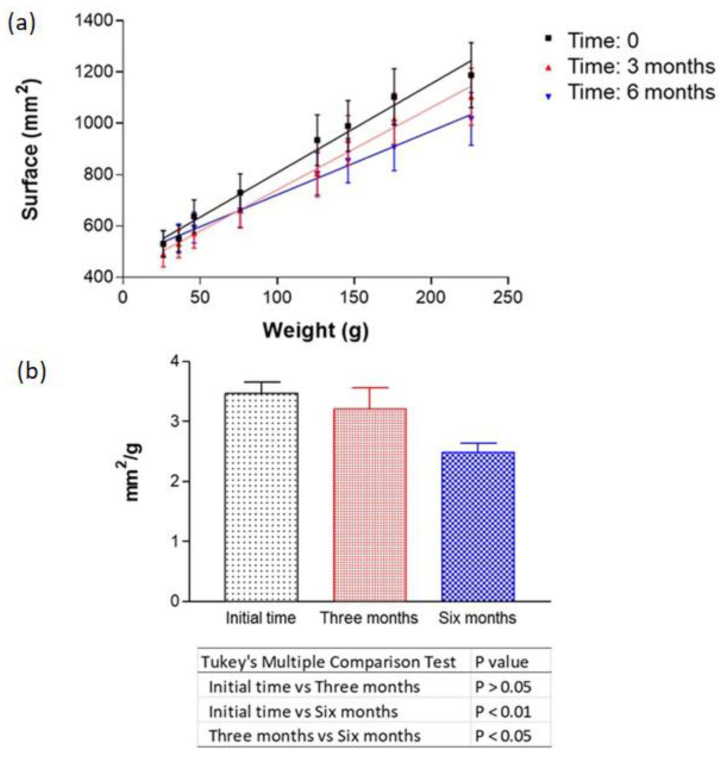
Extensibility of MD-CM-gel over 6 months. (**a**) Kinetic model for MD-CM-gel. (**b**) Statistical parameters.

Figure 4 shows the extensibility of the MD-CM-gel as a function of weight. The extensibility was uniform and there were no changes over time. Statistical analysis showed significant differences between the initial time versus 3 and 6 months.

### 3.3. Droplet Size Determination

**Figure 5:**
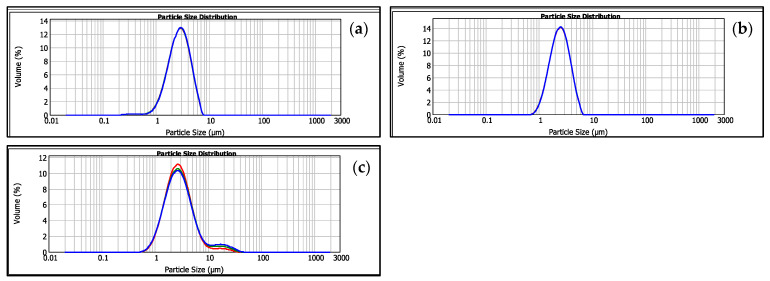
The droplet size of the MD-CM-gel. (**a**) time 0, (**b**) time 3 months, and (**c**) time 6 months.

The droplet size of the MD-CM-gel was measured using the Mastersizer^®^ 2000. Figure 5 shows the distribution and uniform droplet for the emulgel over 6 months of its preparation, showing a particle size of approximately 3 µm. The measurement at 6 months showed a slight increase in the polydisperse index parameter.

### 3.4. Rheology

**Figure 6:**
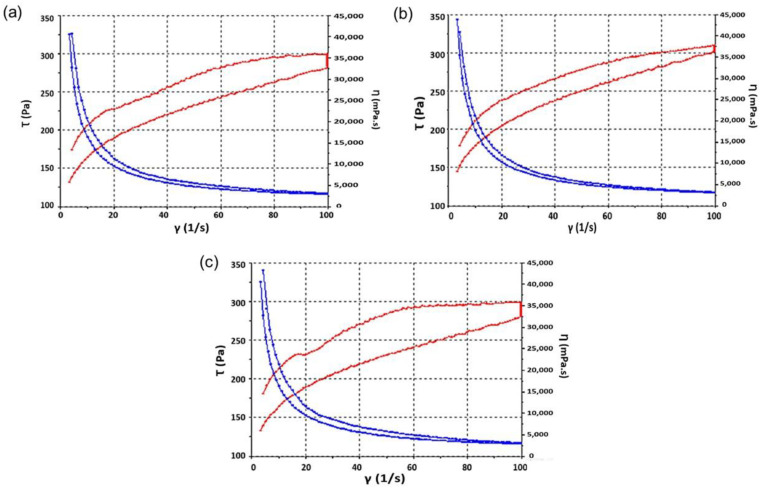
Rheograms of MD-CM-gel at (**a**) time 0 (**b**) 3 months and (**c**) 6 months after preparation (the red line corresponds to the flow curve (τ) while the blue line represents the viscosity curve (η)).

The steady shear properties of the MD-CM-gel are shown in Figure 6. Measurements showed that formulations displayed non-Newtonian behaviour. The formulations exhibited pseudoplastic flow and shear thinning behaviour since the viscosity decreased with an increase in the shear rate from 0.1 to 100 s^−1^. Figure 6 shows the rheograms for the MD-CM-gel freshly prepared, 3 months and 6 months. Although there was a decrease in the viscosity, the extensibility of the emulgel remained unchanged for 6 months, and the decrease in the viscosity had no relevant effect on the spreadability of the emulgel. As shown in Figure 6, viscosity values are 3052 ± 23.59, 2879 ± 48.56, and 2901 ± 43.50 mPa.s for 0, 3, and 6 months, respectively (Table 4). The thixotropic loop was formed between the ascending and descending curves and the area of the loop represented the energy required to break the system structure. The mathematical model that best fit the experimental data was the Cross equation, which describes a general model for pseudoplastic materials, Equation (7):

(7) $$\mathrm{Cross}\mathrm{Equation}\;{{\tau =\dot{\gamma }\cdot {(\eta }_{\infty }+{(\eta }_{0}-\eta_{\infty })/\;1+(\dot{\gamma }/\dot{\gamma }}_{0})}^{n})\;$$

where τ is the shear stress (Pa), $\dot{\gamma }$ is the shear rate (1/s), $\dot{\gamma }$_0_ is the zero shear rate (1/s), $\eta_{0}$ is the zero shear rate viscosity (Pa.s), $\eta_{\infty }\;$is the infinity shear rate viscosity (Pa.s), n is the flow index.

### 3.5. Fourier Transform Infrared Spectroscopy

**Figure 7:**
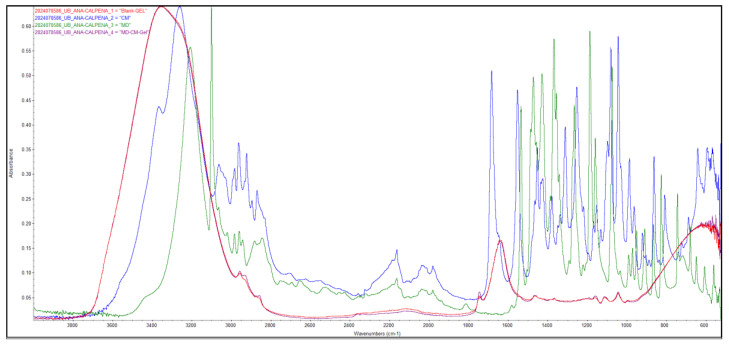
FTIR spectra of the MD-CM-gel, the blank emulgel and the drugs, MD and CM.

The FTIR analysis was conducted to study the interactions between the active ingredients and the excipients composing the emulgel. Figure 7 depicts the spectra of the MD-CM-gel, the blank emulgel, and the pure drugs MD and CM.

**Figure 8:**
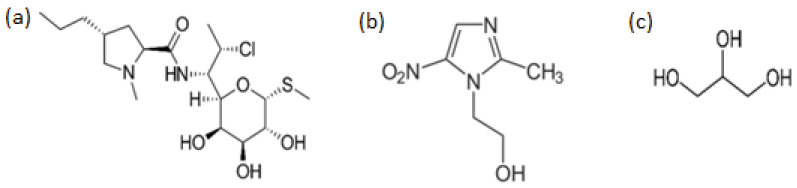
Chemical structure of (**a**) CM, (**b**) MD and (**c**) Glycerine.

CM (Figure 8a) is a very strong basic compound (based on its pKa) and MD (Figure 8b) is a weak base that appears to dissolve maximally at approximate pH ≤ 2.0; at high pH values, the drug has low solubility in water. Simulgel^®^ 600 is composed of acrylamide/sodium acryloyldimethyltaurate copolymer, isohexadecane, and Polysorbate 80. According to the FTIR spectra, the -CONH carbonyl-amide group for CM is not significantly affected. However, the -OH groups of the MD are overlapped by the formulation. Therefore, no interactions with the active ingredients of this pharmaceutical formula occurred. Concerning the rest of the excipients of the emulgel, medium-chain triglycerides (MCTs) are a liquid oil manufactured through the esterification of glycerol using medium-chain fatty acids (caproic (6 C), caprylic (8 C); capric (10 C) and Lauric (12 C)) isolated from natural sources (coconut oil, palm oil, or a combination of the two). It contains carboxylic acids, which are weak acids esterified with glycerine that will not alter the activity of the active ingredients. They can help facilitate the solubility of these compounds. Isohexadecane, whose molecular formula is C16H34 and mass is 226.441 g/mol, is a saturated hydrocarbon with lipophilic properties. No interactions with the active ingredients of this pharmaceutical formula were observed either. Glycerine (Figure 8c) can establish hydrogen bond interactions with polar compounds that have hydroxyl groups or analogues.

Phenonip^®^ is a mixture of phenoxyethanol (≥50%), methylparaben (10–25%), propylparaben (5–10%), and ethylparaben (1–5%). It constitutes a broad bacteriostatic antimicrobial agent spectrum comprising a synergistic mixture of acid esters parahydroxybenzoic acid (parabens) in phenoxyethanol designed for the preservation of a wide range of cosmetics. Phenonip^®^ presents antibacterial activity against Gram+ and Gram- bacteria, yeasts, and molds. No interactions with the active ingredients or excipients are expected.

Concerning possible interactions of the active substances with excipients, CM is a stable base that could form salts with carboxylic acids, but in this formulation, there are no acids to form salts. CM has three hydroxyl groups, which can form hydrogen bonds with water and/or glycerol, favouring solubility. These interactions do not affect the therapeutic activity or the analytical assays of clindamycin. The fatty acids of MCT are esterified with the glycerol that accompanies them. Concerning MD, which is a very weak base, no significant interactions with the excipients of the pharmaceutical formulation are expected.

### 3.6. In Vitro Drug Release Test

**Figure 9:**
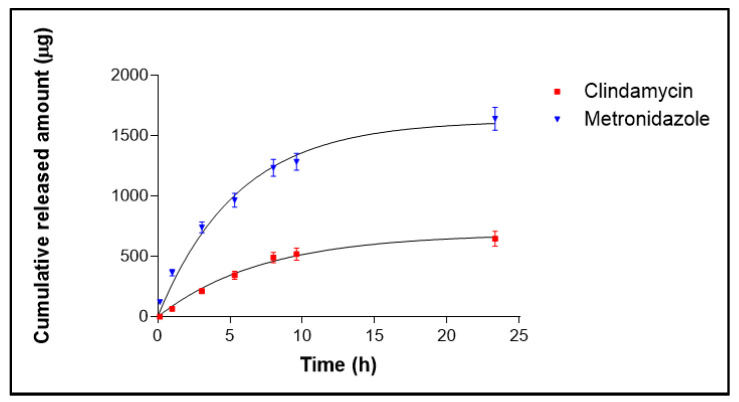
Release profiles of CM and MD from the emulgel formulation. Both profiles fit the one-phase exponential association model.

Figure 9 shows the release profile for CM and MD from the emulgel. The drugs are released in a sustained way up to nearly 24 h, with a faster release in the earlier times. Both drugs best fit the one-phase exponential association. Table 5 shows the parameter values for both drugs.

### 3.7. Ex Vivo Permeation Test

**Figure 10:**
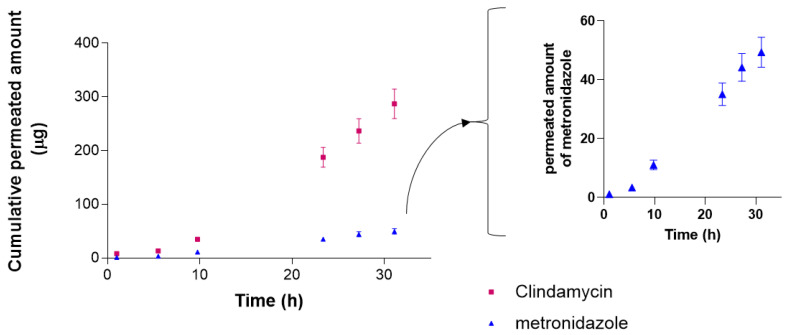
Permeation profiles of CM and MD through human skin from the emulgel formulation. The **right** panel shows the permeation profile for MD on a smaller scale for better visualization of the data.

The permeation study was conducted on ex vivo human skin under an infinite-dose approach. The permeation profiles of the drugs through human skin are depicted in Figure 10. The permeation parameters for both drugs are shown in Table 6.

CM presents a 10-fold flux related to MD. Nevertheless, the lag time for CM is nearly twice the one for MD, resulting in a higher permeability coefficient (*Kp*) for CM. In both drugs, the parameter P2 is higher than P1 indicating that the driving force of the permeation is mainly influenced by the diffusion capacity of the drugs in permeating the skin rather than the partition coefficient vehicle-skin; this fact is especially remarkable for MD, as P2 is about 50-fold higher than P1. Concerning the drug amount retained in the skin, MD is more retained than CM.

### 3.8. Results for Antimicrobial Activity

#### 3.8.1. Interaction Between Antimicrobials

CM showed a lower MIC compared to MD (Table 7). When the two antimicrobials were combined, the MIC decreased by at least one dilution for both MD and CM compared to their MIC separately. This decrease in MIC occurred for all the strains studied.

#### 3.8.2. Antimicrobial Activity of the Formulation

**Figure 11:**
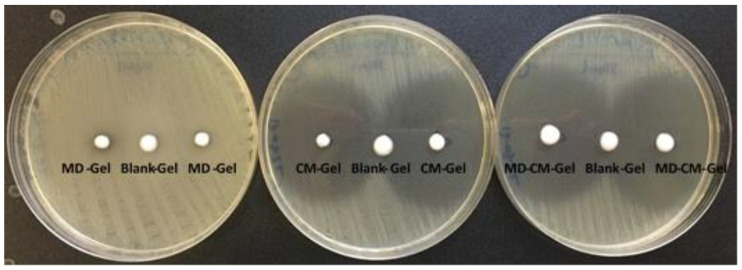
Inhibition halos generated on *C.tuberculostearicum* CECT 763. MD-gel: formulation with metronidazole; CM-gel: formulation with clindamycin; MD-CM-gel: formulation with MD and CM; Blank-gel: formulation without antimicrobial.

The two formulations, MD-CM-gel and CM-gel with clindamycin alone, show clear antimicrobial activity with similar or slightly higher areas of inhibition in the combination of the two products (Table 8). On the other hand, the formulation with only MD, MD-gel, has little or no activity on the microorganisms studied, but when combined with CM, it does not interfere or else facilitate a slight increase in the area of inhibition. The formulation without antibiotic, white gel, does not produce any type of inhibition on the microorganisms studied, as can be seen in Figure 11.

**Figure 12:**
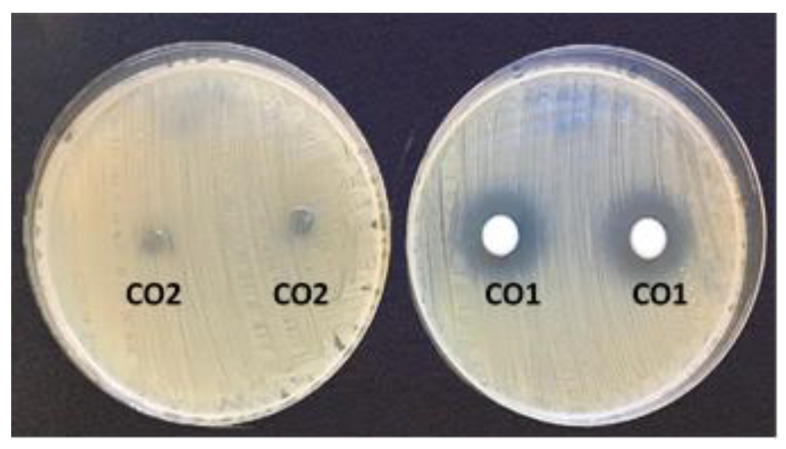
Inhibition halos of commercial Formulations for *C.tuberculostearicum* CECT 763. CO1; formulation with 1% clindamycin. CO2; commercial formulation with 0.75% metronidazole.

Table 9 shows the inhibition halos originated by the solid commercial formulations. The CO1 formulation with clindamycin has activity on the strains studied, but the areas of inhibition are somewhat lower than those of our formulation with the two antimicrobials. However, the solid formulation with only MD inhibits little or no growth in strains (Figure 12).

### 3.9. Toxicity Assay

**Figure 13:**
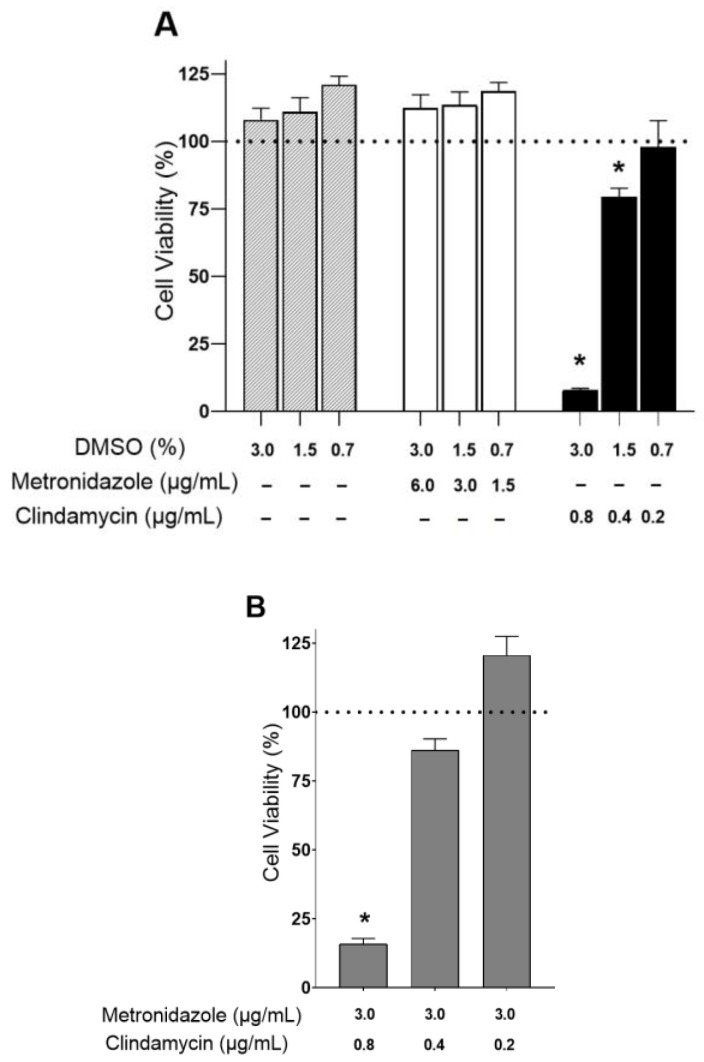
Cell viability of HaCaT keratinocytes after incubation for 24 h with the indicated compounds as single treatment (**A**) or in combination (**B**). To discard the toxicity effects of the vehicle (DMSO), cells were treated with DMSO at the final concentrations used to prepare the CM solutions. Cell viability was assayed by the MTT reduction method; 100% viability was set with the values obtained with the untreated control cells (indicated by a dotted line). Values represent the Mean ± SD (n = 3). Statistical analysis one-way ANOVA Tukey‘s Multiple Comparison Test, * *p* < 0.05 versus control.

The cytotoxicity of MD and CM was evaluated on HaCaT cells after a 24 h incubation at the concentrations indicated in Figure 13A. Results showed that MD was not cytotoxic at concentrations ranging from 1.5 to 6 µg/mL, as cell viability was kept close to the untreated control cells. In contrast, CM caused a significant cytotoxic effect at the higher concentration tested (0.8 µg/mL). At this concentration, cell viability was 10% compared to untreated control cells. At the lowest dose (0.2 µg/mL), cell viability was approximately 100%. In this scenario, we decided to analyse whether the presence of MD could potentiate CM cytotoxicity. For this study, we established experimental conditions to select the MD dose at 3 µg/mL and CM at increasing concentrations. The MTT assay revealed that MD, at the concentration used, did not exert any effect on CM cytotoxicity since cell viability, after 24 h incubation, remained close to that observed with CM alone, at the same concentrations (Figure 13B).

### 3.10. Inflammation Model and Quantification of IL-6 by ELISA

**Figure 14:**
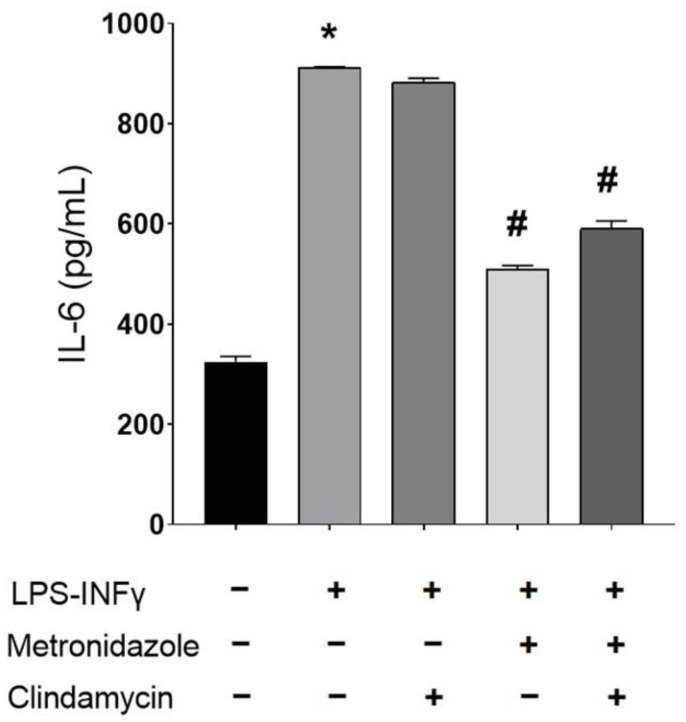
Anti-inflammatory action of MD in the absence and in the presence of CM in the LPS-INFγ inflammation model. HaCat cells were incubated with MD at 0.3 µg/mL, in the absence or presence of CM at 0.2 µg/mL for 3 h and then, challenged with LPS-IFNγ at 0.5 µg/mL and 0.02 µg/mL, respectively. In parallel, cells were incubated in the absence of LPS-IFNγ as a control. Following 48 h incubation, IL6 secreted levels were measured by ELISA. Data are representative of 3 independent experiments. * *p* < 0.05 vs. untreated control cells; # *p* < 0.05 vs. LPS-INFγ treated cells.

Since MD has anti-inflammatory effects at the topic levels and CM is an antibiotic used to treat certain bacterial infections affecting the skin, it was decided to analyse if CM could influence the anti-inflammatory effects of MD. Taking into consideration the cytotoxicity results, this analysis was performed with CM (0.2 µg/mL) and MD (0.3 µg/mL) using the in vitro LPS-IFNγ model of inflammation. Inflammation was evaluated by determining the IL-6 secreted levels by ELISA (Figure 14). The results showed that MD at 0.3 µg/mL significantly reduced, by more than 50%, the IL-6 levels induced by LPS-IFNγ, and CM at 0.2 µg/mL did not significantly interfere with its anti-inflammatory effect.

## 4. Discussion

Rosacea is a chronic inflammatory skin condition characterised by facial redness, papules, and pustules. Topical treatments are often preferred due to their localised action and reduced systemic side effects. There are various topical formulations containing MD or CM used to treat rosacea [26,27,28,29,30]. However, to our knowledge, there is no specific mention of a combined topical formulation containing both MD and CM for this condition. However, both agents are individually effective in treating rosacea, with MD being well-studied and commonly used. Based on the benefits of combined therapies combining MD and oral antibiotics [14,31,32], a topical formulation combining MD and CM was developed. The excipient selection of the emulgel formulations was based on non-comedogenic nor occlusive properties since the skin in rosacea patients is generally sensitive and intolerant [33,34]. Glycerine provides smoothness and has moisturising and lubricant properties. The oily phase consisted of four ingredients combined in Simulgel^®^ 600, which is an emulsifying base with excellent skin tolerability. The addition of water leads to the formation of a stable polymeric network that retains the oily components within its structure. The medium-chain triglycerides exert an emollient action and provide good extensibility of the emulgel [35]. Isohexadecane also acts as an emollient and skin conditioner. The formulation contains Phenonip^®^ to prevent fungal and bacterial contamination. Silicone emulsions (W/S), which do not contain lipids, are also considered good alternatives to the marketed products for the treatment of rosacea [36].

In this study, the MD-CM-gel demonstrated ideal organoleptic properties for dermal application, offering excellent homogeneity, particle size, and consistency, with a pleasant texture and ease of use. The pH values remained stable at 5.7, 5.8, and 5.7 over 6 months, confirming the formulation’s biocompatibility with the skin and ensuring it would not cause irritation. Rheological analysis revealed valuable insights regarding the ease of application, sensory characteristics, and dispensing properties, while also influencing biopharmaceutical factors like drug release rates. The MD-CM-gel displayed a pseudoplastic behaviour with an average viscosity of 2944 ± 38.55 mPa.s at 25 °C [37].

FTIR analysis was performed to identify the interactions between MD-CM-gel, the blank emulgel, and the pure drugs MD and CM. According to the analysis of the spectra of each formulation (Figure 7), there was no evidence of the existence of a covalent bond between the elements that constituted the emulgel. These results are what can be expected in line with those set forth by other authors [38,39]. In vitro drug release studies are conducted to investigate how the drugs are released from the formulation and are available to the skin. The tests allow us to refine and optimize the formulations. The study revealed that CM was released in a significantly lower amount compared to MD. This could be indicative of the different molecular interactions within the emulgel matrix [40,41]. MD exhibits higher water solubility (5.92 mg/mL) compared to CM (3.1 mg/mL) [42,43], facilitating faster diffusion from the emulgel matrix into the aqueous release medium [44]. MD’s pKa values (2.57 and 15.42) promote ionization at pH~7, enhancing its aqueous solubility and contributing to faster release, while CM, with a pKa of 7.6, is less ionized [45]. Additionally, smaller molecules, like MD (molecular weight 171.96 g/mol), diffuse more easily, leading to faster release, whereas larger molecules, like CM (molecular weight 424.98 g/mol), face more resistance [46]. Lipophilicity also impacts release [47]; MD, being more hydrophilic (log P = −0.02), is more water-soluble and releases faster, whereas CM, being more lipophilic (log P = 2.16), tends to interact more with lipid components, slowing its release. These physicochemical properties explain the differences in the release profiles of MD and CM from the emulgel.

A lower release of CM does not imply therapeutic failure, as long as enough of the drug reaches the target. The slower release may even be beneficial, reducing systemic absorption and minimising the risk of adverse effects like antibiotic resistance or toxicity. In contrast, the faster release of MD supports its anti-inflammatory role, offering more immediate symptom relief. The complementary release profiles of CM and MD work synergistically, addressing both microbial and inflammatory aspects of rosacea effectively. Microbiological studies also showed synergy between CM and MD, with a significant reduction in minimum inhibitory concentration (MIC) and larger inhibition halos compared to individual drugs. These findings suggest the combined formulation may enhance efficacy against rosacea-associated microorganisms. However, further preclinical and clinical studies are needed to confirm these benefits and understand the combination’s full potential in clinical practice.

The mathematical modelling of these release profiles enables us to describe the behaviour of these drugs during the release process. The one-phase association kinetic model best described the release of the drugs, suggesting a rapid initial release followed by a plateau, a pattern often desired in topical applications for sustained effect [24]. Despite the lower release amount of CM, the release rate was only slightly slower than MD.

Ex vivo permeation tests are conducted using human or animal skin and provide insights into the absorption, penetration, and retention of topical formulations, which is valuable in the development of effective dermatological treatments. By understanding how active ingredients behave, researchers can optimise drug delivery systems and ensure safety and efficacy before clinical trials. This approach not only accelerates the research and development process but also reduces the reliance on animal testing, aligning with the ethical standards of modern pharmacology [48]. In our study, we observed a greater flux for CM compared to MD, which is 6.5-fold higher, suggesting that CM has a more efficient transdermal transport rate. This is further supported by the permeability coefficient (*kp*) being approximately three times higher for CM, indicating a more pronounced tendency for CM to penetrate the skin barrier. However, the lag time for CM is twice that of MD, which implies that, while CM permeates more effectively once initiated, its onset is slower. This could be attributed to the physicochemical properties of CM. Physicochemical properties, such as lipophilicity, melting point, molecular weight, Log P, and solubility of the drugs, impact the permeation capacity [49,50]. The stark contrast of *P1* between DM and MD indicates that CM presents higher affinity. *Elewski* investigated the percutaneous absorption of MD in different vehicles and concentrations. The study found that the vehicle significantly affects the percutaneous absorption of the MD and cream formulations showed the highest penetration, followed by lotions and gels [51].

In both cases, the diffusion coefficient (*P*2) was greater than *P*1, especially for MD, indicating that the driving force behind the permeation is mainly due to the physicochemical properties of the drugs. The similar *P*2 values for both drugs indicate that their molecular mobility within the skin is comparable. Yet, the difference in the amount retained in the skin points to a greater affinity of MD for the skin tissue than for the receptor fluid, which could explain the higher retention of MD in the skin despite its lower permeation. This suggests that MD may be more localized in the skin, potentially leading to a local therapeutic effect but less systemic absorption. The potential local effect is supported by the predicted plasma concentration that would be achieved at the steady state after the application of the emulgel to the skin: the estimated *Css* for both drugs would be much below those plasma concentrations observed in the oral route of administration suggesting that the application of the emulgel via the cutaneous route would not cause systemic side effects, hence, the emulgel would be a safe dosage form [52,53].

Microbiological assays have established that the combination of CM and MD does not counteract each other but even enhances the antimicrobial activity of CM. From a microbiological point of view, the efficacy of the formulation is superior to commercial formulations with CM alone or MD alone. Since there is an overgrowth of certain microorganisms in skin with rosacea and inflammatory processes [54], the formulation with MD and CM can help in both processes. CM can reduce the overgrowth of microorganisms found on the skin and MD can reduce inflammation, as seen in other studies [55]. This combination of effects explains the reduction in symptoms of the formulation. The combination of the two active ingredients facilitates the application of the treatment for rosacea disease.

Moreover, the HaCaT cell line is commonly used for in vitro toxicity assessment of dermal formulations before pre-clinical testing on animal models. This is due to their ease of cultivation, rapid growth, and high sensitivity to toxic agents, similar to other cell lines [56]. In this study, the results confirmed that MD was not cytotoxic at concentrations ranging from 1.5 to 6.0 µg/mL, and CM at 0.2 µg/mL did not induce significant cytotoxic effects on HaCaT cells, demonstrating their good biocompatibility with human keratinocytes [57].

Cytokines are regulatory proteins that play a key role in inflammatory responses [58]. Interleukin-6 (IL-6) is a significant pro-inflammatory cytokine involved in the pathogenesis of rosacea. Elevated levels of IL-6 have been observed in patients with rosacea, indicating its role in the inflammatory processes associated with the condition [59]. IL-6 contributes to the immune response, inflammation, and angiogenesis, which are key factors in the development and severity of rosacea [60]. Inflammation was evaluated by measuring IL-6 secretion levels using ELISA (Figure 14). The findings revealed that MD at 0.3 µg/mL significantly reduced IL-6 levels induced by LPS-IFNγ by over 50%. In contrast, CM at 0.2 µg/mL did not significantly alter its anti-inflammatory effect [61]. Metronidazole’s ability to modulate IL-6 in rosacea is likely due to its broader anti-inflammatory and antimicrobial properties. In rosacea patients, the increased levels of IL-6 correlate with ROS production and activation of the hypoxia-induced factor HIF-1α [59,62], and both effects are mediated through the IL-6/JAK/STAT3 pathway [63,64]. By reducing IL-6 levels, metronidazole may contribute to a decrease in the oxidative stress response and the associated symptoms of rosacea.

## 5. Conclusions

The development of an emulgel formulation combining MD and CM showed promising results as a possible treatment for rosacea. The formulation exhibited appropriate physicochemical and biopharmaceutical properties and sustained drug release. Additionally, it demonstrated adequate permeability through human skin and showed no toxicity in a HaCaT cell model. Antimicrobial studies revealed that the combination of MD and CM does not exhibit antagonism and is more effective than commercial treatments or antibiotics used separately. MD-CM-gel is effective against the most frequent bacterial strains found on the human sebaceous skin. Moreover, the MD and CM did not induce significant cytotoxic effects on HaCaT cells and the efficacy assessment, based on IL-6 secretion levels, demonstrated that MD significantly reduced IL-6 levels induced by LPS-IFNγ by more than 50%.

The properties of the dual-active emulgel formulation represent an advance in the prevention and treatment of rosacea and may facilitate compliance in patients with this skin condition, as only a single application would be required. These results suggest that MD-CM emulgel may be an effective alternative for the treatment of rosacea, pending further preclinical and clinical studies.

## Figures and Tables

**Table 1:** Composition of the emulgel formulation containing MD and CM.

Compound	Quantity
MD	0.75%
CM	1%
Simulgel^®^ 600	4.5%
MCT ^1^	10%
Isohexadecane	5%
Glycerine	5%
Phenonip	0.3%
Purified water	q.s.p. 73.45%

^1^ MCT = medium chain triglycerides.

**Table 2:** Analytical conditions for the determination of MD and CM.

Parameter	MD Condition			CM Condition		
Wavelength (nm)	318			210		
Flow (mL/min)	1.0			1.0		
Injection volume (µL)	50			50		
Chromatography time	20			20		
Retention time	7			7.2		
Eluent A	0.01 M KH_2_PO_4_, pH = 3.0			0.025 M KH_2_PO_4_, pH = 7.5		
Eluent B	MeOH; ACN			ACN		
Eluent C	MeOH			-		
Gradient						
	Time (min)	%B	%C	Time (min)	%B	
	0.0	6	8	0.0	45	
	7.5	6	8	8.0	45	
	9.0	18	24	9.0	70	
	13.0	18	24	13.0	70	
	14.5	6	8	14.0	45	
	20.0	6	8	20.0	45	

KH_2_PO_4_: Potassium dihydrogen phosphate; MeOH: methanol; ACN: acetonitrile.

**Table 3:** MD-CM-gel pH values for over 6 months.

Time	0	3 Months	6 Months
pH	5.7 ± 0.1	5.8 ± 0.1	5.7 ± 0.1

**Table 4:** Data analysis for the rheograms of the MD-CM-gel at time 0, 3 months, and 6 months of preparation.

Time (Months)	Viscosity Value at 100 s^−1^ (mPa.s)	Flow	Model Fitted	Ramp (Up/Down)	Thixotropy (Pa/s)
0	3052 ± 23.59	Shear thinning	Cross	0.9992/0.9999	23,470
3	2879 ± 48.56	Shear thinning	Cross	0.9887/0.9997	21,520
6	2901 ± 43.50	Shear thinning	Cross	0.9945/0.9998	21,770

**Table 5:** Drug release parameters from the emulgel according to a one-phase exponential association. Values represent Means ± standard error (n = 6).

Best-Fit Values	Clindamycin	Metronidazole
Ymax (µg)	689.1 ± 28.42	1628.0 ± 66.77
K (h^−1^)	0.1387 ± 0.0125	0.1813 ± 0.0182
Half-time (h)	4.998	3.824
R^2^	0.9975	0.9954

Ymax = total amount of drug released; K = release rate constant.

**Table 6:** Permeation parameters: flux (*Js*), lag time (*Tl*), permeability coefficient (*Kp*), partition (*P1*), and diffusion (*P2*) coefficients drug amount retained in the skin (*Qret*) and plasma concentration at the steady-state (*Css*) of CM and MD after 31 h of in vitro permeation tests in human skin. The results are given in median (minimum and maximum).

	Clindamycin	Metronidazole
Jss (µg/h)	18.44	2.84
	(18.07–19.53)	(2.20–2.97)
Tl (h)	7.00	3.64
	(6.14–8.41)	(3.14–4.85)
Kp (cm/h)	0.00184	0.00038
	(0.00100–0.00214)	(0.00025–0.00052)
P1 (cm)	0.03717	0.000828
	(0.02264–0.04854)	(0.00019–0.000914)
P2 (1/h)	0.0496	0.0457
	(0.0391–0.0511)	(0.0365–0.0601)
Qret (µg/cm^2^)	6.46	13.34
	(5.68–7.32)	(10.61–15.51)
Css (ng/mL)	43.65	20.88

**Table 7:** MIC results in µg/mL of CM and MD separately and in combination for each of the strains.

Microorganism	MIC Alone		MIC in Combination	
	MIC CM	MIC MD	MIC CM	MIC MD
*Corynebacterium simulans* DSM 44415	2.44	117.19	1.22	58.59
*Corynebacterium tuberculostearicum* CECT 763	0.61	937.5	0.31	468.75
*Cutibacterium acnes* ATCC 6919	0.07	468.75	0.009	58.6
*Escherichia coli* ATCC 25922	19.53	1875	9.77	937.5
*Micrococcus luteus* ATCC 10240	9.77	3750	1.22	937.5
*Staphylococcus aureus* ATCC 29213	0.15	937.5	0.0175	234.38
*Staphylococcus capitis* CECT 7101^T^	0.15	468.75	0.07	234.38
*Staphylococcus epidermidis* ATCC 12228	0.15	468.75	0.07	234.38
*Staphylococcus hominis* CECT 234^T^	0.15	468.75	0.07	234.38
*Streptococcus mitis* ATCC 49456	0.035	234.38	0.009	58.59

**Table 8:** Diameter in mm of the inhibition halos originated by the different formulations: CM-gel: formulation with CM; MD-gel: formulation with metronidazole; MD-CM-gel: formulation with MD and CM.

Strains	CM-gel	MD-gel	MD-CM-gel
*Corynebacterium simulans* DSM 44415	39 ± 1	13 ± 0	45 ± 2
*Corynebacterium tuberculostearicum* CECT 763	44 ± 0	0	48 ± 0
*Cutibacterium acnes* ATCC 6919	75 ± 2	11 ± 0	70 ± 2
*Escherichia coli* ATCC 25922	20 ± 0	0	18 ± 1
*Micrococcus luteus* ATCC 10240	38 ± 1	0	43 ± 1
*Staphylococcus aureus* ATCC 29213	31 ± 1	8 ± 0	34 ± 0
*Staphylococcus capitis* CECT 7101^T^	39 ± 1	0	40 ± 1
*Staphylococcus epidermidis* ATCC 12228	39 ± 0	14 ± 1	39 ± 0
*Staphylococcus hominis* CECT 234^T^	38 ± 2	6	42 ± 1

**Table 9:** Inhibition halos in mm of commercial solid formulations. CO1; Formulation with 1% clindamycin. CO2; Formulation with 0.75% metronidazole.

Strains	CO1	CO2
*Corynebacterium simulans* DSM 44415	16	0
*Corynebacterium tuberculostearicum* CECT 763	20	0
*Cutibacterium acnes* ATCC 6919	50	0
*Escherichia coli* ATCC 25922	0	0
*Micrococcus luteus* ATCC 10240	22	7
*Staphylococcus aureus* ATCC 29213	25	0
*Staphylococcus capitis* CECT 7101^T^	24	0
*Staphylococcus epidermidis* ATCC 12228	27	0
*Staphylococcus hominis* CECT 234^T^	24	0

## Data Availability

The original contributions presented in this study are included in the article.
